# CtcS, a MarR family regulator, regulates chlortetracycline biosynthesis

**DOI:** 10.1186/s12866-019-1670-9

**Published:** 2019-12-10

**Authors:** Lingxin Kong, Jia Liu, Xiaoqing Zheng, Zixin Deng, Delin You

**Affiliations:** 1 State Key Laboratory of Microbial Metabolism, Joint International Research Laboratory of Metabolic and Developmental Sciences, and School of Life Sciences & Biotechnology, Shanghai Jiao Tong University, Shanghai, 200030 China; 2 Department of Immunology, Hebei Medical University, Shijiazhuang, 050017 Hebei China

**Keywords:** MarR family regulator, Tetracycline family antibiotics, Chlortetracycline, CtcS, Transcriptional regulation

## Abstract

**Background:**

Chlortetracycline (CTC) is one of the commercially important tetracyclines (TCs) family product and is mainly produced by *Streptomyces*. CTC is still in a great demand due to its broad-spectrum activity against pathogens. Engineering transcriptional control allows the cell to allocate its valuable resources towards protein production and provides an important method for the build-up of desired metabolites. Despite extensive efforts concerning transcriptional regulation for increasing the productivities of TCs, the regulatory mechanisms of the CTC biosynthesis remain poorly understood.

**Results:**

In this study, the possible regulatory function of CtcS, a potential member of MarR (multiple antibiotic resistance regulator) family of transcriptional regulators in *S. aureofaciens* F3, was demonstrated. Knockdown of *ctcS* altered the transcription of several biosynthesis-related genes and reduced the production of tetracycline (TC) and CTC, without obvious effect on morphological differentiation and cell growth. Especially, CtcS directly repressed the transcription of the adjacent divergent gene *ctcR* (which encodes a putative TC resistance efflux protein). A CtcS-binding site was identified within the promoter region of *ctcR* by DNase I footprinting and an inverted repeat (5′-CTTGTC-3′) composed of two 6-nt half sites in the protected region was found. Moreover, both CTC and TC could attenuate the binding activity of CtcS with target DNA.

**Conclusion:**

*ctcS* regulated the production of TC and CTC in *S. aureofaciens* F3 and the overexpression of it could be used as a simple approach for the construction of engineering strain with higher productivity. Meanwhile, CtcS was characterized as a TC- and CTC-responsive MarR family regulator. This study provides a previously unrecognized function of CtcS and will benefit the research on the regulatory machinery of the MarR family regulators.

## Background

Tetracyclines (TCs) designate an important family of compounds widely used in pharmaceutical industry, confined animal feeding operations and aquaculture [1]. As protein biosynthesis inhibitors, TCs could chelate divalent cations and competitively bind to the 30S ribosomal subunit, blocking the aminoacyl-tRNA entering into the aminoacyl (A)-site [2]. Besides the well-documented broad-spectrum activity against pathogenic bacteria, many TC derivatives show antiparasitic activities [1], like oxytetracycline (OTC) and doxycycline, which even exhibited inhibitory effect on human matrix metalloproteinases [3, 4]. As one of the important members of TCs, CTC was firstly isolated from *Streptomyces aureofaciens* in 1948 [5]. It has been used as drug for the treatment of eye infections, fowl typhoid and pullorum disease, and is mainly used in animal husbandry. Up to now, these compounds have been industrially mass-produced and the exploration of more effective and potent routes for construction of high-yield strains is still a growing field of recent studies.

It is known that the production of natural products in *Streptomyces* is usually regulated by multiple regulatory proteins for controlling metabolic flux, in respond to internal physiological and environmental conditions [6, 7]. Different families of transcriptional regulators have been demonstrated to be involved in the control of antibiotic production. The TetR family transcriptional regulator DepR1 positively regulated the daptomycin production in the industrial producer *S. roseosporus* SW0702 [8]. The *Streptomyces* antibiotic regulatory protein (SARP) family regulator NosP activated the transcription of structural genes for nosiheptide biosynthesis [9] and responded to both peptidyl and small-molecule ligands derived from the precursor peptide [10]. Recently, MarR family transcriptional regulators have been identified in antibiotic biosynthesis gene cluster. MarR proteins commonly have a triangular-shaped structure with a dimerization domain and a winged helix-turn-helix DNA binding domain. Generally, the conventional regulatory mechanism of MarR proteins entails a divergently encoded regulated gene. The MarR family transcriptional regulator DptR3 activated daptomycin biosynthesis and morphological differentiation in *S. roseosporus* [11]. Actually, several regulators for TCs production have been identified. Actually, several regulators for TCs production have been reported. The SARP regulator OtcR was an efficient pathway specific activator of OTC biosynthesis in *S. rimosus* M4018. The deletion of *otcR* completely abolished OTC production and the tandem expression of two copies under the control of strong SF14 promoter increased OTC production to more than six times [11]. OtcR was found to activate the transcription of *oxy* genes through direct interaction with the conversed 9-nt direct repeats [11]. Ctc11, the homologous protein of OtcR was reported to activate the expression of *oxy* cluster in heterologous host *S*. *coelicolor* CH999 [11] and *Streptomyces lividans* K4–114 [12]. Moreover, the LAL (LuxR) family transcriptional regulator OtcG has been identified in the OTC biosynthetic gene cluster (*otc* cluster) in *S. rimosus* [13]. Inactivation of *otcG* reduced OTC biosynthesis by more than 40%, however the overexpression of it by introducing a second copy under the constitutive promoter ermE*p didn’t influence the final OTC yield significantly [13]. So, OtcG was proved playing ‘conditionally-positive’ role in OTC production*.* Taking the reported phosphate-mediated control of OTC production into account, a more complex ‘fine tuning’ role of OtcG in overall expression of genes for OTC biosynthesis was envisaged [13]. However, the utilization of transcriptional control engineering for high yield strain constructions is largely dependent on the elucidation of the regulatory system, which in the case of CTC is still lacking.

The study of CTC biosynthesis began with the identification of biosynthetic genes [14, 15]. However, the biosynthetic pathway of CTC was intricately elucidated, due to the unknown genetic differences between *S*. *aureofaciens* wild type and random mutant strains. The biosynthetic gene cluster of CTC (*ctc* cluster) in industrial strain *S. aureofaciens* F3 has been identified previously and the halogenase CtcP has been proved responsible for the transformation of TC to CTC. Strikingly, the overexpression of *ctcP* has contributed to the productivity improvement of CTC [16]. Even with this success, the recent study is still focused on the exploration of more effective and potent routes to the construction of high-yield strains. In order to explore the biosynthetic regulatory mechanism and provide insight into future synthetic engineering construction of CTC, the regulatory role of CtcS was characterized in this study. The bioinformatic analysis of CtcS suggested that it is a possible MarR family transcriptional regulator. Genetic interruption and complementation of *ctcS* proved its positive role in regulating TC and CTC production. And the overexpression of *ctcS* resulted in little improvement of TC and CTC yield. Meanwhile, the target CtcS-regulated genes were identified and the CtcS-binding sequence was determined by DNase I footprinting. Moreover, both TC and CTC attenuated the binding activity of CtcS with the target DNA. These findings suggested that when integrated with other metabolic engineering strategies, the manipulation of *ctcS* might be used for the construction of high-yield strain.

## Results

### *ctcS* encodes a putative MarR family transcriptional regulator

**Fig. 1:**
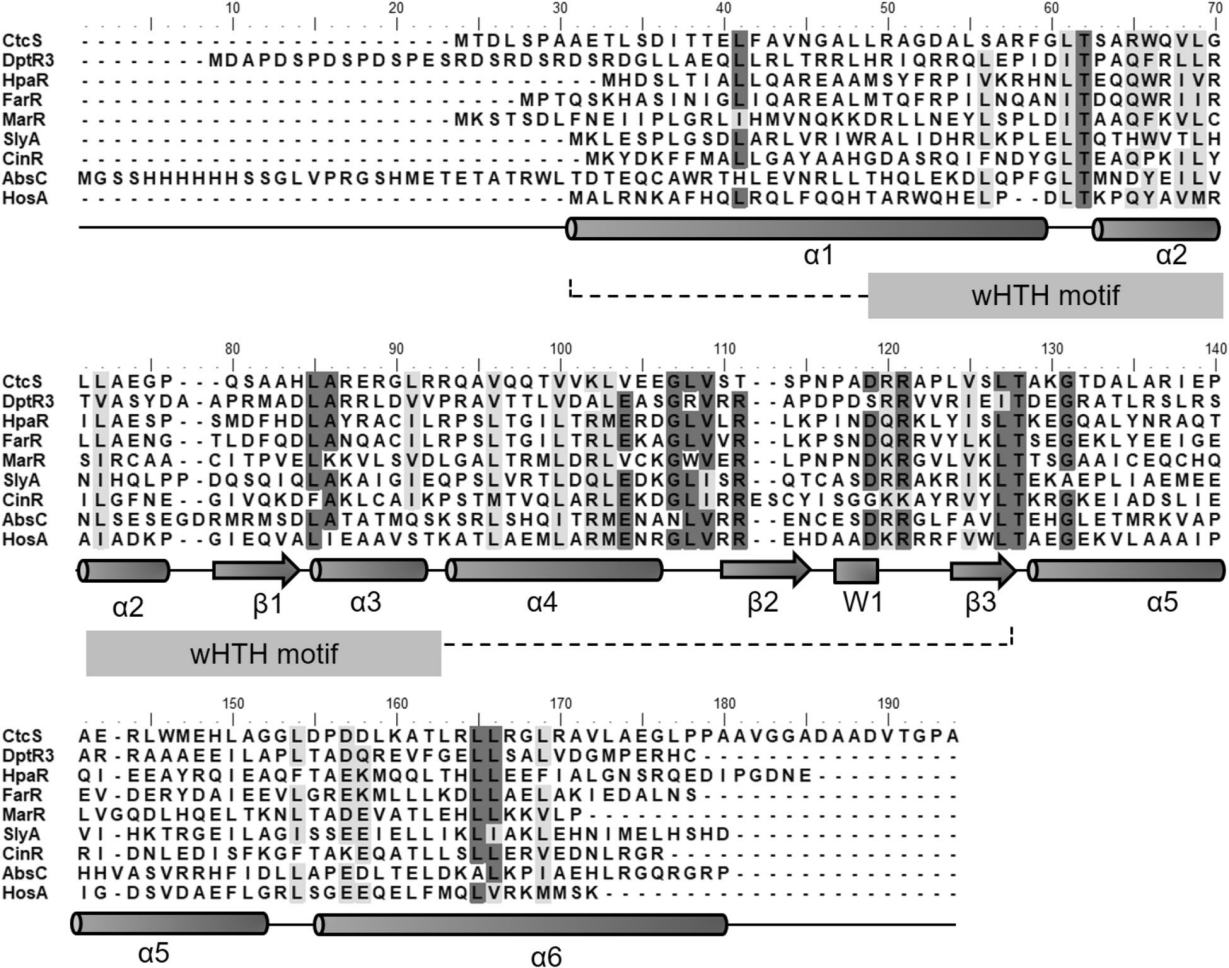
Multiple sequence alignment of CtcS with proteins of the MarR family. The alignment was generated using ClustalX. Light and dark shading indicated ≥70% similarity and identity at that position, respectively. Secondary structure elements indicated below the alignment showed conservation of the wHTH motif and are based on the MarR crystal structure (PDB: 1JGS), with α-helices represented as cylinders, β-strands as arrows and the wing as a filled box. The conserved wHTH motif was underlined by dotted line

The *ctcS* gene contains 498 nucleotides (nt) and encodes a 165-amino-acid putative MarR family transcriptional regulator with a conserved helix-turn-helix (HTH) DNA-binding motif homologous to MarR [17] (Fig. 1). The divergently transcribed gene *ctcR* is located upstream of *ctcS* and encodes a putative TC resistance efflux protein. The nucleotide sequences and deduced amino acid sequences of *ctcR*-*S* are highly homologous to those of *otrB*-*R* involved in the OTC biosynthesis in *S. rimosus.* CtcS exhibits 55% identity with OtrR (OxyTA1) and CtcR exhibited 60% identity to OtrB*.* The OtrR and the promoter region of *otrB* (*otrBp*) have been selected for the construction of inducible expression system (Potr*) for aromatic polyketide [18]. However, the in situ role of OtrR in regulating OTC production has not been elucidated in depth. The arrangements of *ctcR*-*S* and *otrB*-*R* are similar to that has been found in *dptR3*-*orf16*. The gene *dptR3* encoded a MarR regulator DptR3 and *orf16* encoded a putative ABC transporter ATP-binding protein. The deletion of *dptR3* reduced daptomycin production significantly and delayed aerial mycelium formation and sporulation on solid media [11]. DptR3 was found to stimulate daptomycin production indirectly by altering the transcription of structural genes for daptomycin biosynthesis. Meanwhile, DptR3 activated the transcription of its own gene *dptR3* but repressed the transcription of *orf16* [11]*.* Other MarR proteins have been reported responsible for the regulation of antibiotic biosynthesis, such as PenR and PntR for phenalinolactone biosynthesis [19]. The secondary structure of CtcS was analyzed by PSIPRED as is shown in Fig. 1. The proposed DNA binding domains of CtcS was depicted following other MarR proteins and adopted the conserved winged helix (or winged helix-turn-helix, wHTH) fold [20] (Fig. 1), which is defined topologically by secondary structure elements arranged as α1-β1-α2-α3-β2-W1-β3. The sequence spanning α2 through α3 constitutes the general HTH motif, with α3 being the most invariable DNA recognition helix [20]. Taken together, these data suggested that CtcS might function as a MarR family transcriptional regulator of CTC biosynthesis.

### CtcS positively regulates the production of TC and CTC

**Fig. 2:**
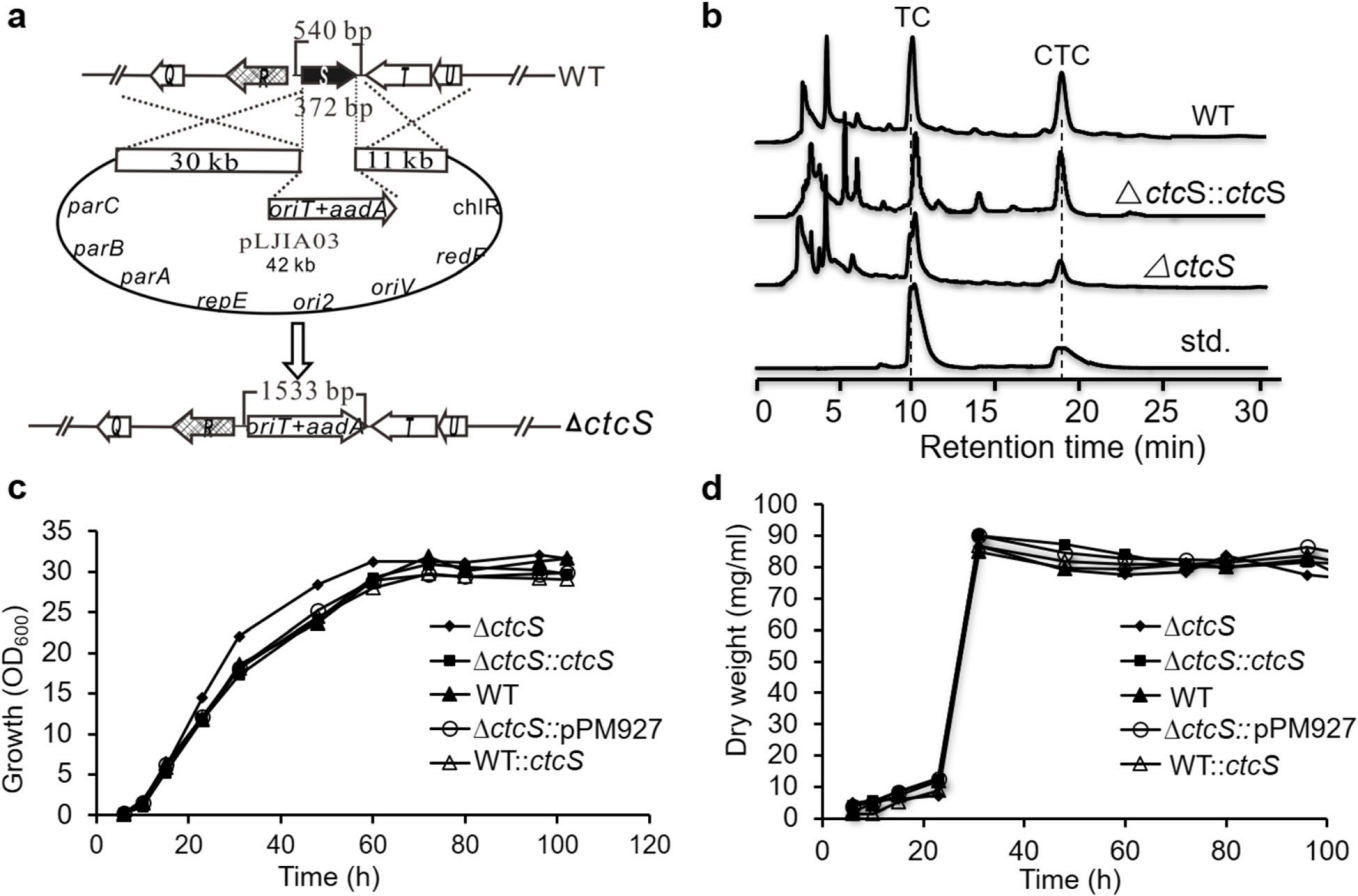
The construction and phenotypic characterization of mutant strains. **a** Schematic construction of *∆ctcS* mutant. **b** HPLC profile of CTC and TC accumulation in different strains. **c** Growth curve of *∆ctcS* mutant and WT strains. **d** Cell growth was measured in cell dry weight. Three clones were selected and the error bars showed the standard deviation of three independent experiments of the selected clones in b, c and d

**Fig. 3:**
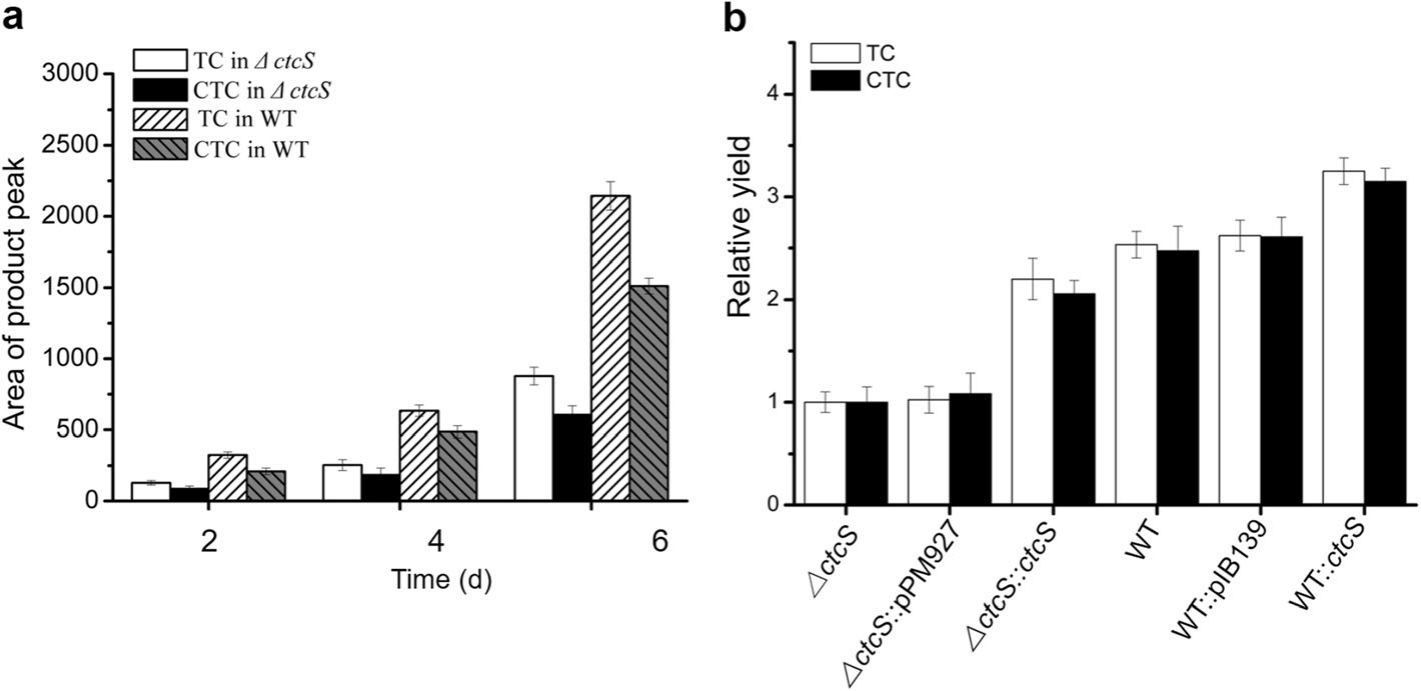
Analysis of CTC and TC production in different strains. **a** Time-course analysis of TC and CTC production in *∆ctcS* and WT strains. **b** Quantitative analysis of TC and CTC production in *∆ctcS, ∆ctcS*::pPM927, *∆ctcS*::*ctcS*, WT, WT::pIB139 and WT::*ctcS* strains. For comparison, the yield in *∆ctcS* strain are determined as 1. Error bars showed the standard deviation of three independent experiments

To elucidate the role of *ctcS* in CTC biosynthesis, 372 bp of *ctcS* was replaced by spectinomycin resistance gene by homologous recombination following the PCR targeting-based gene disruption protocol [21, 22] (Fig. 2a), and the disruption of *ctcS* was verified by PCR (Additional file 1: Figure S1). Then the fermentation products in *ΔctcS* strain were analyzed by high-performance liquid chromatography (HPLC). As can be seen from Fig. 2b, the production of TC and CTC declined in *ΔctcS* strain. To demonstrate that these reductions were solely due to the deletion of *ctcS*, the *ctcS*-complementary strain *ΔctcS*::*ctcS* (Additional file 1: Figure S1) was constructed by integrating one copy of intact *ctcS* gene under the control of erythromycin resistance gene promoter (ermE*p) on the plasmid pPM927 [23]. The production of TC and CTC in *ΔctcS*::*ctcS* strain increased, compared with that of the *ΔctcS* strain. The deletion of *ctcS* did not show obvious effect on the formation of aerial mycelia and sporulation on the solid SFM medium. To validate that the productivity changes were only induced by the regulatory role of *ctcS*, both of the growth curve and biomass were characterized in the WT, *ΔctcS* and *ΔctcS*::*ctcS* strains (Fig. 2c,d). Consistently, these strains shared similar characters and exhibited negligible differences. For the quantitative comparison of the productivities, time course analysis was conducted in the *ΔctcS* and WT strains (Fig. 3a). During the whole fermentation process, the accumulation of TC and CTC was smaller in *ΔctcS* mutant than that in the WT strain. Subsequently, further quantitative estimation of the productivities was conducted (Fig. 3b). The yield of TC and CTC in *ΔctcS* was only 40% of the WT strain, and the production in the *ΔctcS*::*ctcS* was about 80% of the WT strain (Fig. 3b), after deducting the negligible productivity change exerted by empty plasmid in *ΔctcS*::pPM927. These findings indicated that *ctcS* indeed regulated the TC and CTC biosynthesis. To further consolidate the findings, the pIB139 [16] derivative plasmid pLJIA15 carrying intact *ctcS* gene was integrated into the genome of WT strain, resulting in the overexpressing strain WT::*ctcS*. HPLC analysis of the fermentation products showed that the yields of TC and CTC in *ctcS*::*ctcS* strain was about 1.3 and 1.2 times of the WT strain, respectively. Meanwhile, the referred strain WT::pIB139 produced nearly the same amounts of products as that in WT strain (Fig. 3b). Taken together, the *ctcS* positively regulated the production of TC and CTC. This suggested an efficient approach for the engineering construction of high-yield strains, when combined with other metabolic engineering strategies.

### CtcS affects gene transcription in *ctc* cluster

**Fig. 4:**
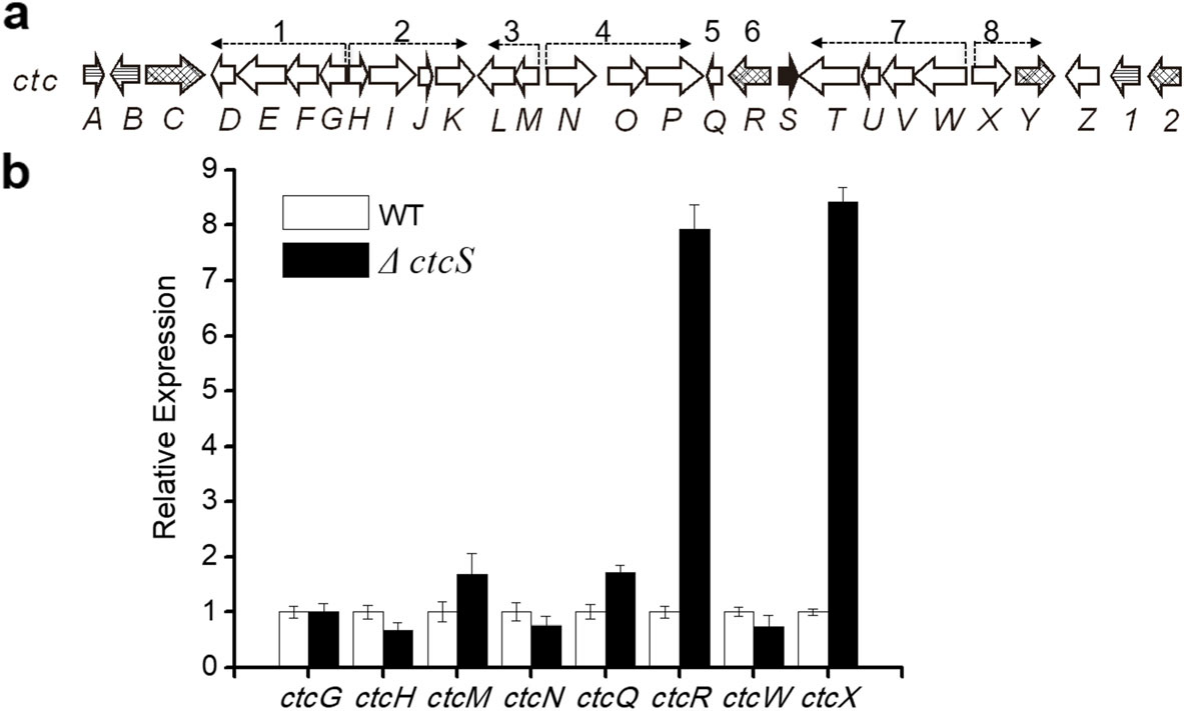
Transcriptional analysis of genes in the WT and *∆ctcS* strains. **a** Organization of the operons encoded by *ctc* cluster. The detected transcription units were marked with arabic numerals. **b** RT-qPCR analysis of transcription levels in *∆ctcS* mutant and WT strain. The relative transcription levels of each gene were obtained after normalization against the internal reference *hrdB*. Error bars showed the standard deviation of three independent experiments

To further elucidate the regulatory role of CtcS in CTC biosynthesis, the real-time quantitative PCR (RT-qPCR) assay of the transcripts encoded by *ctc* cluster was performed. As the genes necessary for CTC structural assembly within the *ctc* cluster have been grouped into seven small transcription units (*ctcG*-*D*, *ctcH*-*K*, *ctcM*-*L*, *ctcN*-*P*, *ctcQ, ctcT*-*W*, and *ctcX*-*Y*) previously [24] (Fig. 4a)*,* the first gene of each operon was selected as representative during the transcription analysis. The RT-qPCR was performed with RNAs isolated from the WT and *∆ctcS* strains grown in fermentation medium for 2 days (at which time CTC has been synthesized referred to Fig. 3a), respectively. From the data depicted in Fig. 4b, the transcription level of most of the operons was similar to that in the WT strain. While, the increased transcriptions of *ctcM* and *ctcQ* in *∆ctcS* strain were negligible when compared with that of WT strain (Fig. 4b). The *ctcR* transcription in *∆ctcS* strain was 8-fold higher than that in WT strain (Fig. 4b), which was consistent with the previously reported regulatory role of *dptR3* on *orf16* [11]. Surprisingly, the transcription levels of genes *ctcX-Y* also increased in *∆ctcS* (Fig. 4b), indicating that CtcS may exerted repression effect on *ctcX-Y* either directly or indirectly.

### CtcS specifically binds to the bidirectional *ctcR*-*ctcS* promoter region

**Fig. 5:**
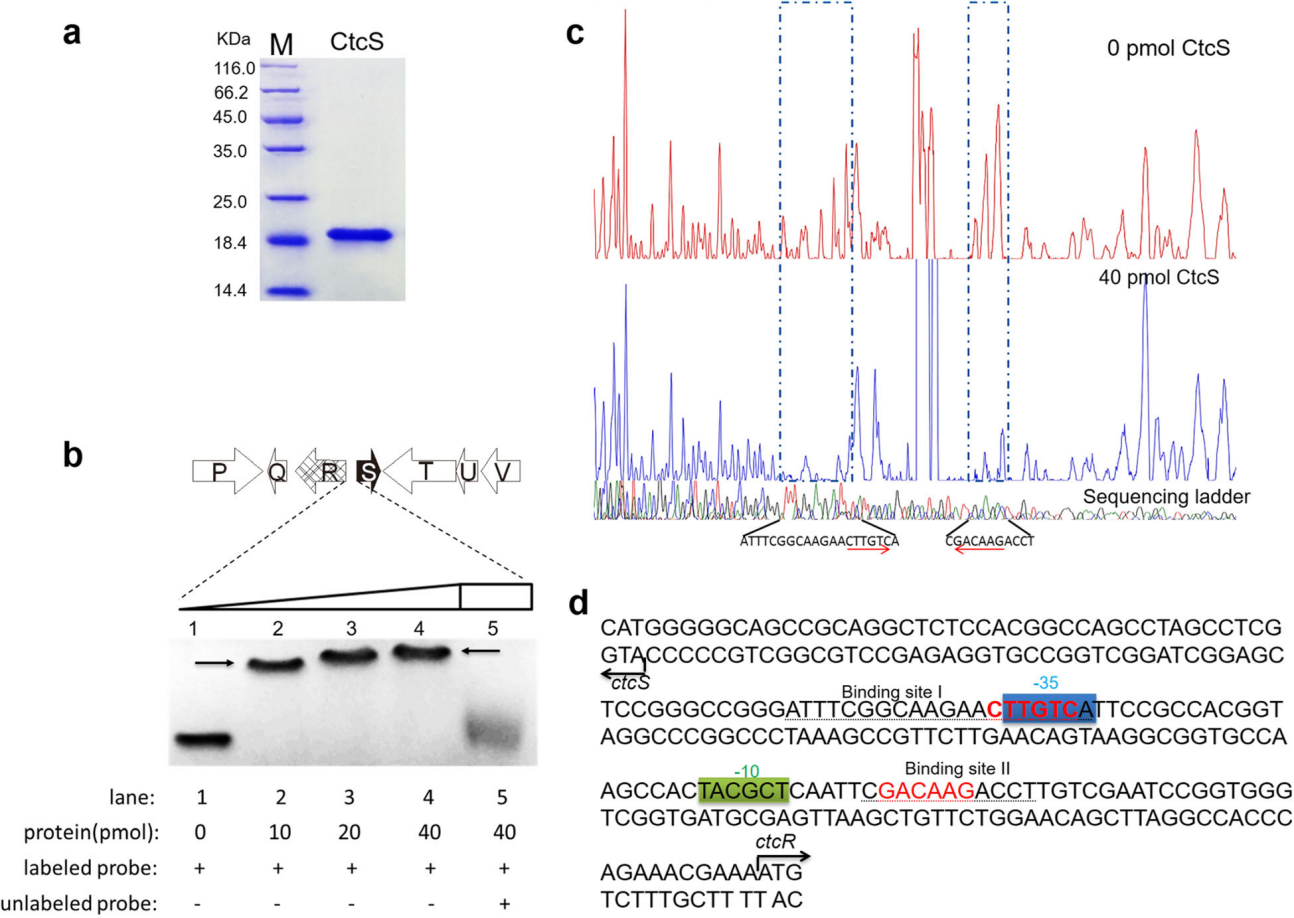
DNA-binding properties of CtcS targeting the *ctcR* promoter region. **a** Purified CtcS analyzed by SDS-PAGE. **b** EMSAs of CtcS binding to the *ctcS*-*ctcR* intergenic region. The 148 bp FAM-labelled DNA fragment of the intergenic region was incubated with increasing concentrations of CtcS protein (lanes 2–4; lanes contain 10, 20, 40 pmol CtcS, respectively). Lane 1, negative control without CtcS; lane 5, 40 pmol CtcS with labeled and unlabeled probes. The shifted bands are indicated by arrows. **c** DNase I footprinting of CtcS in the *ctcS*-*ctcR* intergenic region. The sequence around the protected region is indicated below the electrophoregrams, and the palindromic sequence of the protected region is indicated with red arrows. **d** Nucleotide sequence of *ctcS*-*ctcR* intergenic region. The two CtcS-binding sites are underlined and the direct repeats are marked with red in bold. The bent arrows indicate the transcription orientation of *ctcS* and *ctcR*, and the possible − 10 and − 35 regions are indicated with green and blue box, respectively

Typically, MarR proteins bind the palindromic sequences within the intergenic region between the *marR* gene and a divergently oriented gene (or operon) as dimers [20]. To determine whether *ctcS* affect the expression of *ctcR* through direct interaction with DNA, the *ctcS* gene was firstly expressed in *E. coli* BL21(DE3)/pLysE and then was purified as His_6_-tagged recombinant CtcS. The purity of the resultant protein was detected by sodium dodecyl sulfate-polyacrylamide gel electrophoresis (SDS-PAGE) analysis (Fig. 5a). The calculated molecular weight (MW) of His_6_-tagged CtcS subunit is 19.3 kDa, which is consistent with that observed by SDS-PAGE. As many MarR family regulators have been reported to act as a dimer [25], the CtcS protein was then transferred to size exclusion chromatography analysis (Additional file 2: Figure S2). The CtcS showed a peak with similar retention time to the standard ovalbumin (Molecular Weight is 44 kD) and obviously different from that of lysozyme (MW is 14 kD). This data suggested that CtcS exist in the form of a dimer. To determine whether CtcS directly modulate the gene mentioned above, the electrophoretic mobility shift assay (EMSA) experiment was performed according to the protocol described before [26]. As can be seen from Fig. 5b, the purified His_6_-CtcS was observed to bind to the *ctcS*-*ctcR* intergenic region in a concentration-dependent manner and generated significantly shifted bands. Our findings indicated that CtcS directly repress the transcription of *ctcR* through interaction with the promoter region of it. To uncover the precise binding sequence of CtcS, DNase I footprinting assay was conducted with the same FAM-labeled probe, in the presence or absence of His_6_-CtcS protein. Two protected regions were found on the coding strand of *ctcR* (Fig. 5c), overlapping the potential − 10 and − 35 regions of *ctcR* promoter (Fig. 5d). Further analysis of the sequence within these two sites revealed one inverted repeat comprised of two 6-nt half sites: binding site I 5′-ATTTCGGCAAGAACTTGTCA-3′ and binding site II 5′-CGACAAGACCT-3′ (Fig. 5d). Our findings indicated that CtcS may directly affect the transcription of the adjacent gene *ctcR* by blocking the access of RNA polymerase to its promoter region.

### TC and CTC attenuate the DNA-binding activity of CtcS

**Fig. 6:**
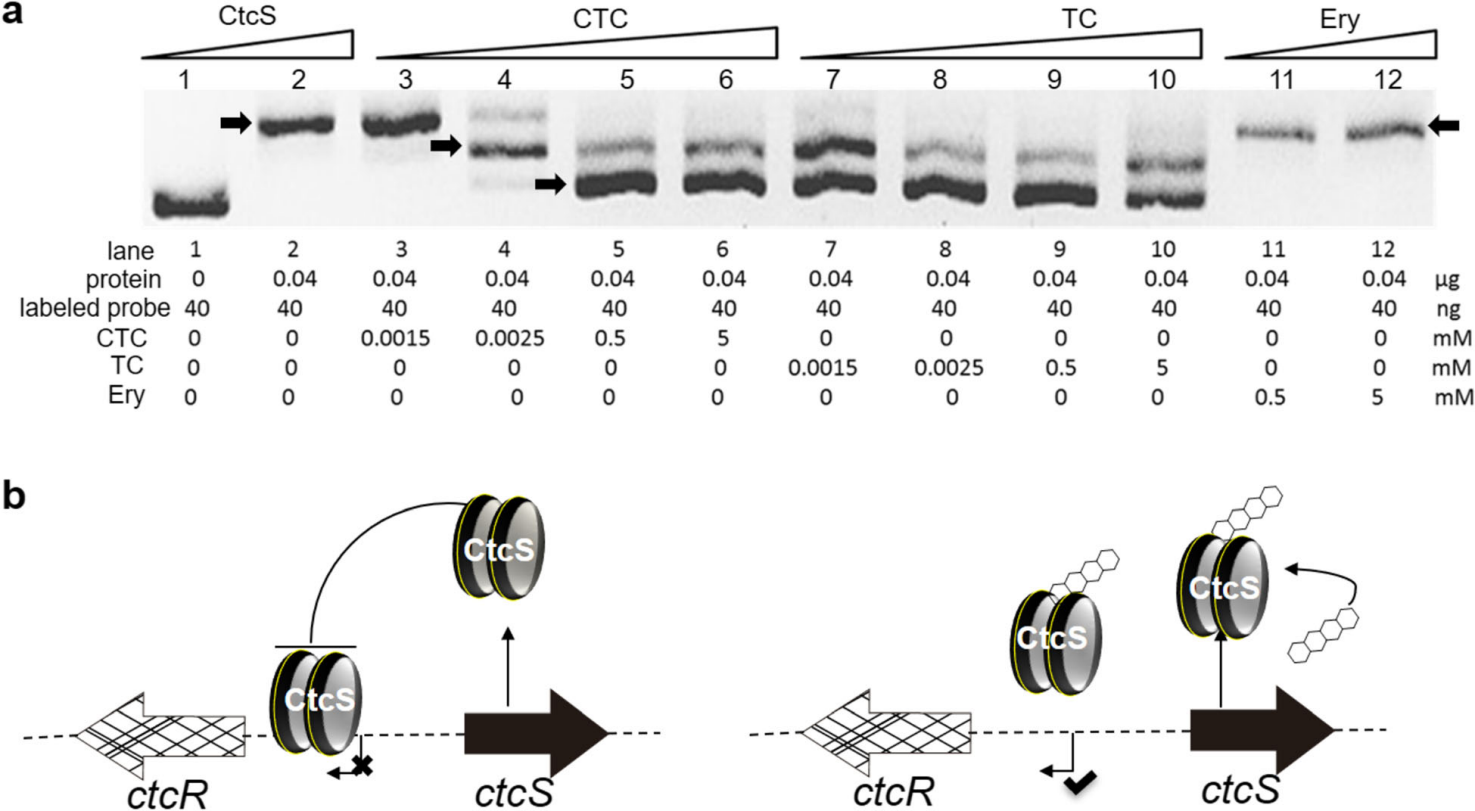
Derepression effect of TC and CTC. **a** EMSA analysis of the binding affinity of CtcS for *ctcR*-*ctcS* interaction fragment with the presence of CTC and TC, and the erythromycin (Ery) was used as control. **b** Schematic diagram of the CtcS-regulated expression of *ctcR* affected by biosynthesized molecules. The four fused hexagons denoted TC and CTC molecules, the large black arrow indicated the binding of CtcS with TC and CTC, and the curving black line indicated the movement of translated CtcS toward target DNA binding sites

Many MarR proteins have been demonstrated to act both as activators by either ligand-induced relieve of transcriptional repression and as repressors through competition with an activator or RNA polymerase (RNAP) for the same binding site [27]. It has been reported that such transcriptional regulation can be triggered by conformational changes upon the binding of small-molecule ligands to MarR proteins [25]. However, DptR3, the MarR family positive regulator in daptomycin biosynthesis, did not show any affinity for daptomycin [11]. Since CtcS has been proved to regulate the TC/CTC production, we tried to determine whether TC/CTC could act as ligand of CtcS and affect its binding activity. In order to test this possibility, TC and CTC of increasing concentrations were added into the complex system of CtcS and the abovementioned labeled probe (Fig. 6a). Erythromycin (Ery), which is structurally different from TCs, was used as negative control. From the EMSA data shown in Fig. 6a, the DNA-binding affinity of CtcS would be decreased by the presence of TC and CTC, and this effect also occurred in a concentration-dependent manner. Especially, TC seemed to be a more effective ligand than CTC, as the addition of CTC at 1.5 μM showed no effect on the DNA-protein complex while TC with the same concentration could lead to the dissociation of the complex, leading to another weakly shifted band near the free probe (Fig. 6a). Moreover, only when the concentration of CTC was up to 0.5 mM, could it result in the same changed shift bands with that exerted by 2.5 μM of TC. These results demonstrated that the biosynthesized TC and CTC were able to attenuate the binding activity of CtcS with its target DNA (Fig. 6a).

## Discussion

*Streptomyces* species are renowned for the ability to produce diverse bioactive secondary metabolites [28]. The produced secondary metabolites supply a chemical diversity that greatly exceeds compounds synthesized chemically and have been pre-selected through millions of years of evolution to interact effectively with biological targets [29]. The production of those secondary metabolites is typically under stringent control of a complex regulation system. Transcription regulation is critical to correctly interpret the environmental signals and translate them into appropriate transcriptional responses to allocate its cellular resources towards the production of desired metabolites [7, 30]. transcriptional control engineering requires careful control over titrating protein levels and assembling biological components in new ways to produce systems with practical applications in synthetic biology [7]. Recently, genetic manipulation of regulatory genes has emerged as an important tool for construction of high-yield strains [6, 31–33].

To date, regulators located in gene cluster encoding OTC biosynthesis have been identified, such as OtcR [11], Ctc11 [34] and OtcG [13]. All these regulators have been proved to directly regulate OTC production, and could be developed for high yield strain constructions through transcriptional control engineering. Compared to the regulators for OTC production, little is known for the regulators of CTC production. Informatics analysis pinpointed CtcS as a potential MarR family regulator with the typical wHTH motif (Fig. 1). MarR family regulators are widespread in prokaryotes, and members of MarR family of transcription regulators exhibit high structural similarity despite low sequence similarity [35]. The sequence dissimilarity might be required to respond to diverse signaling molecules and recognize unique DNA targets [35]. To verify the regulatory role, the *ctcS* gene was genetically interrupted firstly. TC and CTC production were reduced in the resultant mutant (Fig. 2). The over-expression of *ctcS* contributed to the relative higher yield of TC/CTC (Fig. 3). These findings suggested the positive regulatory role of CtcS in TC and CTC biosynthesis. For the mechanism dissection, RT-qPCR was performed to identify the regulatory target of *ctcS* in *∆ctcS* strain. According to the data shown in Fig. 4b, the transcription level of *ctcX*-*ctcY* and *ctcR* increased dramatically. CtcX showed 65% sequence identity with OxyE, which was an ancillary but a more efficient nonessential monooxygenase of OxyL for the C4 hydroxylation during OTC biosynthesis [36]. So, the increased transcription of *ctcX* in *∆ctcS* strain may facilitate the hydroxylation at the C-4 position, preventing the glucuronidation and spontaneous oxidation and thus contribute to guaranteeing the cellular metabolism toward TC and CTC biosynthesis [36]. However, the higher transcription of *ctcX* in *∆ctcS* strain could not lead to more accumulation of TC and CTC, as the transcription of genes encoding the enzymes necessary for the assembling of the molecular skeleton was similar with that in WT strain. Of course, other possibilities can’t be excluded that the intermediates modified by CtcX might constitute ligands to allosterically induce conformational changes in other regulators playing a positive role in the biosynthesis of TC/CTC, or there are other regulators of this pathway unknown to interact with TC/CTC or other intermediary biosynthetic products. The regulators of MarR family have been reported to control transcription of several genes including those encodes for multi-substrate transporters for multidrug resistance [35]. Gene *ctcR* is located upstream of *ctcS* and encodes a putative TC resistance efflux protein, which suggest a possible role in detoxification. However, it is hard to say the relationship between the increased transcription of *ctcR* and the altered production. Similarly, based on the study of *orf16*, the alteration of daptomycin production in the *∆dptR3* mutant did not result from varying expression of *orf16*. Other possible unknown DptR3 targets were proposed to affect daptomycin biosynthesis [11]. As the regulation of MarR proteins has been observed to spread across the genome of various organisms, resulting in either cross-talk or competition with other transcriptional regulators [35], other target genes of CtcS-like regulators would be found in the future exploration of the complex regulatory system. A CtcS-binding site was identified within the intergenic region of *ctcS*-*ctcR* possibly by an inverted repeat (5′-CTTGTC-3′) (Fig. 5). According to the conventional regulatory mode of MarR proteins [25], the schematic model of the CtcS-regulated expression of *ctcR* affected by biosynthesized molecules was depicted in Fig. 6b. This layout allows the CtcS to bind specifically to the intergenic region between *ctcS* and *ctcR* to repress the transcription of *ctcR*. On the binding of small molecule ligands such as TC and CTC, the DNA binding activity of CtcS was attenuated, resulting in a relive of repression allowing gene expression.

The MarR family regulators serve physiological roles as sensors of changing environments and is critical for controlling virulence factor production, modulating bacterial response to antibiotic, oxidative stresses and catabolism of environmental aromatic compounds [20]. Nevertheless, the full spectrum of MarR proteins involving in gene regulation has yet to be revealed, in large part because the ligands to which they respond are often unknown [25]. So, identifying the ligands for MarR regulators is critical for the understanding of molecular regulatory mechanisms. Future structure characterization of the ligand-binding pocket within CtcS may provide a much-needed tool toward identifying the ligands of MarR homologs for which the effector remains unknown.

## Conclusion

Transcription regulation is critical for optimizing protein levels and the subsequent cellular levels of metabolites [7]. The regulation of antibiotics biosynthesis has been established as a key aspect of the investigations on the secondary metabolism in *Streptomyces*. Transcription regulation of the secondary metabolism is complex and frequently involves pleotropic global regulators and cluster-situated repressors or activators [29]. Recently, overexpressing or disrupting pleiotropic/pathway-specific regulatory genes has emerged as an efficient metabolic engineering approach to facilitate product development and commercialization [6, 31–33]. We have identified a MarR family regulator and demonstrated its regulatory role in CTC and TC biosynthesis. Meanwhile, both of TC and CTC could attenuate the activity of CtcS for binding the target DNA. Abundant MarR regulators have been found in various organisms and been involved in cross regulation within a complex regulatory system [35]. However, more ligands responsive MarR proteins are still needed for the regulatory machinery illustration of regulators in this family. So, the characterization of CtcS is an important step towards that goal and will allow the construction of more sophisticated systems in the future.

## Methods

### Bacterial strains and growth conditions

Bacterial strains and plasmids used in this study are listed in Additional file 3: Table S1. Primers are listed in Additional file 4: Table S2. General manipulations of *E. coli* and *Streptomyces* were carried out according to the published procedures [21, 37]. SFM medium (per liter contained 2% agar, 2% mannitol, 2% soybean powder, pH 7.2) was used for sporulation. TSBY medium (per liter contained 3% tryptic soy broth, 1% yeast extract, 10.3% sucrose, pH 7.2) was used for mycelium growth. The seed and fermentation medium were the same as used in our previously study [16]. It is worthy to be mentioned that 0.2% potassium bromide was added into the seed medium and 0.25% into the fermentation medium for exclusively TC production previously [16]. In order to monitor the change of TC and CTC production directly exerted by *ctcS*, all the media used during the fermentation process was used without the addition of potassium bromide.

### HPLC analysis of TC and CTC

The fermentation cultures were treated with oxalic acid and then the supernatants were analyzed by Agilent HPLC series 1100 with an Agilent TC-C18 column (5 μm, 4.6 [inside diameter] by 250 mm). The column was equilibrated with 80% (vo/vol) solvent A (20 mM oxalic acid and 20 mM triethylamine in water, pH 2.0) and 20% (vol/vol) solvent B (acetonitrile) and developed with a linear gradient (5–35 min, from 20% B to 55% B, 35–40 min, from 55% B to 80% B) and then kept 100% (vol/vol) B for 5 min at a flow rate of 0.6 mL∙min^− 1^ and UV detection at 360 nm. The time course fermentation and the analysis of the resultant products at different timepoint (2, 4 and 6 d) were conducted according to the described procedure above.

### Protein expression and purification

For the expression, the gene *ctcS* was inserted into the NdeI and EcoRI sites of pET28a, leading to recombinant plasmid pLJIA07. The expression plasmid pLJIA07 was transformed into *E. coli* BL21 (DE3)/pLysE. Cultures were grown in LB medium containing 50 μg/mL kanamycin to OD_600_ of 0.6. 0.2 mM IPTG was added to induce protein expression at 16 °C for 24 h. Cells were suspended in 20 mL lysis buffer (50 mM Tris-HCl, pH 8.0, 0.3 M NaCl), lysed by sonication for 40 min and centrifued 12,500 g for 60 min at 4 °C. The supernatant was used to purify the His_6_-tagged CtcS using Ni^2+^-nitrilotriacetic acid spin column (Qiagen, Germany). The protein was eluted by a linear gradient using buffer 50 mM Tris-HCl, pH 8.0, 0.3 M NaCl 500 mM imidazole. Purified protein was stored in PBS buffer at − 80 °C. The size exclusion chromatography was performed with AKTA FPLC P-920 using superdex 200 10/300 column from GE Healthcare, using ovalbumin and lysozyme (GE Healthcare, China) as control (Additional file 2: Figure S2).

### RNA isolation and RT-qPCR assay

RNA was isolated using the Total RNA Isolation Kit (Beijing SBS Genetech Co., Ltd.) from mycelia of WT and its derivative *∆ctcS* mutant strains grown two days in fermentation medium. RT-qPCR was performed using the Maxima™ SYBR Green qPCR Master Mix (Thermo Fisher Scientific) and the Applied Bio-systems 7500 Fast Real-Time PCR System (Thermo Fisher Scientific) under the following conditions: 5 min at 95 °C followed by 40 cycles of 10 s at 95 °C, 30 s at 60 °C. A final dissociation stage was run to generate a melting curve. The essential *hrdB* gene encoding sigma-like transcription factor was used as the internal reference. Primers used were shown in Additional file 4: Table S2. Data for the RT-qPCR assays were collected from independent triplicate experiments.

### EMSAs and DNase I footprinting assay

The FAM-labeled oligos within the promoter regions of *ctcR* (365 bp) were firstly PCR amplified with 2× TOLO HIFI DNA polymerase premix (TOLO Biotech, Shanghai) using primers M13F-47/M13R-48 (Additional file 4: Table S2) and were then purified by the Wizard® SV Gel and PCR Clean-Up System (Promega, America) and quantified with NanoDrop 2000C (Thermo, America). EMSA was performed in a reaction buffer at the total volume of 20 μL containing 50 mM Tris-HCl (pH 8.0), 100 mM KCl, 2.5 mM MgCl, 0.2 mM DTT, 10% (vol/vol) glycerol with 0.04 pmol FAM-labeled probers at room temperature. Various concentrations of His_6_-tagged CtcS (0, 10, 20, 40 pmol) were added into the system. Meanwhile, sheared salmon sperm DNA was added to a final concentration of 100 ng/μL in the reaction system for the elimination of the non-specific binding. After incubation for 30 min at 25 °C, the reaction system was loaded into a 6% native-PAGE gel buffered with 0.5 × Tris-borate-EDTA buffer. Gels were scanned with the ImageQuant LAS 4000 mini (GE Healthcare, America). The competitive EMSA was performed in a similar 20 μL reaction system with 40 ng probe, 40 ng protein (except the first lane) and varied concentration (0.0015, 0.0025, 0.5, 5 mM) of compounds (TC, CTC and Ery).

DNase I footprinting assays were carried out following the protocol described before [26]. For each assay, the FAM-labeled DNA probes were incubated with different amounts of His_6_-tagged CtcS (0, 40 pmol) in a total volume of 40 μL at 25 °C for 30 min. Subsequently, 10 μL solution containing about 0.015 units DNase I (Promega, America) and 100 nmol freshly prepared CaCl_2_ were added and further incubated at 25 °C for 1 min. The reaction was quenched by the addition of 140 μL DNase I stop solution, which contained 200 mM unbuffered sodium acetate, 30 mM EDTA and 0.15% sodium dodecyl sulfate (vol/vol) (SDS). The system was firstly extracted with phenol/chloroform for the removal of protein, and then was precipitated with ethanol. The resultant precipitation was dissolved in 30 μL MilliQ water (Millipore). The preparation of the DNA ladder, electrophoresis and data analysis were performed according to the procedure described previously [26], except that the GeneScan-LIZ600 size standard (Applied Biosystems, America) was used.

### Growth measurement

Spores were inoculated into TSBY medium with the proportion of 0.1% and cultivated at 30 °C for 3 days. Then, 5 mL seed broth was inoculated into 100 mL fresh TSBY medium and cultivated at 30 °C. 1 mL culture was collected at different time point (0, 6, 10, 15, 23, 31, 48, 60, 72, 80, 96, and 102 h) to monitor the OD_600_ for the depiction of growth curve and another 1 mL culture was centrifuged, washed by MilliQ water and dried at 65 °C for biomass measurement.

### Multiple sequence alignment and secondary structure prediction

Multiple sequence alignment was conducted using BioEdit software and the referred homologous proteins were listed as bellow. DptR3 (GenBank: AAX31530.1) from *Streptomyces filamentosus* NRRL 11379; HpaR (GenBank: ADT77985.1) from *E. coli* W; FarR (UniProtKB/Swiss-Prot: P0DPR8.1) from *Neisseria gonorrhoeae*; MarR (GenBank: AAK21292.1) from *E. coli*; SlyA (GenBank: RWU72049.1) from *Salmonella enterica subsp. enterica* serovar Typhimurium; CinR (GenBank: AAB57775.1) from *Butyrivibrio fibrisolvens*; AbsC (PDB: 3ZMD) from *Streptomyces* ***c****oelicolor*; HosA (NCBI: YP_002413753.2) from *E. coli* UMN026. The prediction of secondary structure of CtcS was conducted by PSIPRED v4.0 (http://bioinf.cs.ucl.ac.uk/psipred/).

## Supplementary information

**Additional file 1: Figure S1.** Construction and verification of the strains used in this study. (**a**) Schematic construction of *∆ctcS::ctcS* strains. (**b**) PCR verification of *∆ctcS* mutants. PCR products using genomic DNA from *∆ctcS* mutants were in three lanes marked 1, 2 and 3. The amplified product of WT strain was used as control. (**c**) PCR verification of *∆ctcS::ctcS* strains and WT::*ctcS* strains. Primers thiof-thior were used for the verification of the existence of plasmid pLJIA13 in *∆ctcS::ctcS* strains (lanes marked 1, 2 and 3) and plasmid pLJIA15 in WT::*ctcS* strains (lanes marked 4, 5 and 6). Both of the genomic DNA of *∆ctcS* strain and WT strain were used as control.

**Additional file 2: Figure S2.** Size exclusion chromatography of His_6_-tagged CtcS. (**a**) Size exclusion chromatography analysis of purified His_6_-tagged CtcS. (**b**) Size exclusion chromatography analysis of standard ovalbumin (1) and lysozyme (2).

**Additional file 3: Table S1.** Bacterial strains and plasmids used in this study.

**Additional file 4: Table S2.** Primers used in this study.

## Data Availability

The sequences for *ctcS* and CtcS were available with the accession number GenBank:HM627755 and GenBank: AEI98662.1, respectively. All data used or analyzed during this study are available from the corresponding author on reasonable request.

## Abbreviations

CTC: Chlortetracycline
EDTA: Ethylene diamine tetraacetic acid
EMSA: Electrophoretic mobility shift assay
Ery: Erythromycin
FAM: 6-carboxyfluorescei
HPLC: high-performance liquid chromatography
HTH: Helix-turn-helix
IPTG: Isopropyl-β-D-1-thiogalactoside
LAL: Large ATP-binding regulators of the LuxR family
MarR: Multiple antibiotic resistance regulator
MW: Molecular weight
OTC: Oxytetracycline
RT-qPCR: Real-time quantitative PCR
SARP: *Streptomyces* antibiotic regulatory protein
SDS-PAGE: Sodium dodecyl sulfate-polyacrylamide gel electrophoresis
TC: Tetracyclines
wHTH: Winged helix-turn-helix
WT: Wild type
