# Supplementary figures and images for: CtcS, a MarR family regulator, regulates chlortetracycline biosynthesis
Source: BMC Microbiol. 2019 Dec 10;19:279. doi: 10.1186/s12866-019-1670-9 (PMC6905112; doi:10.1186/s12866-019-1670-9)

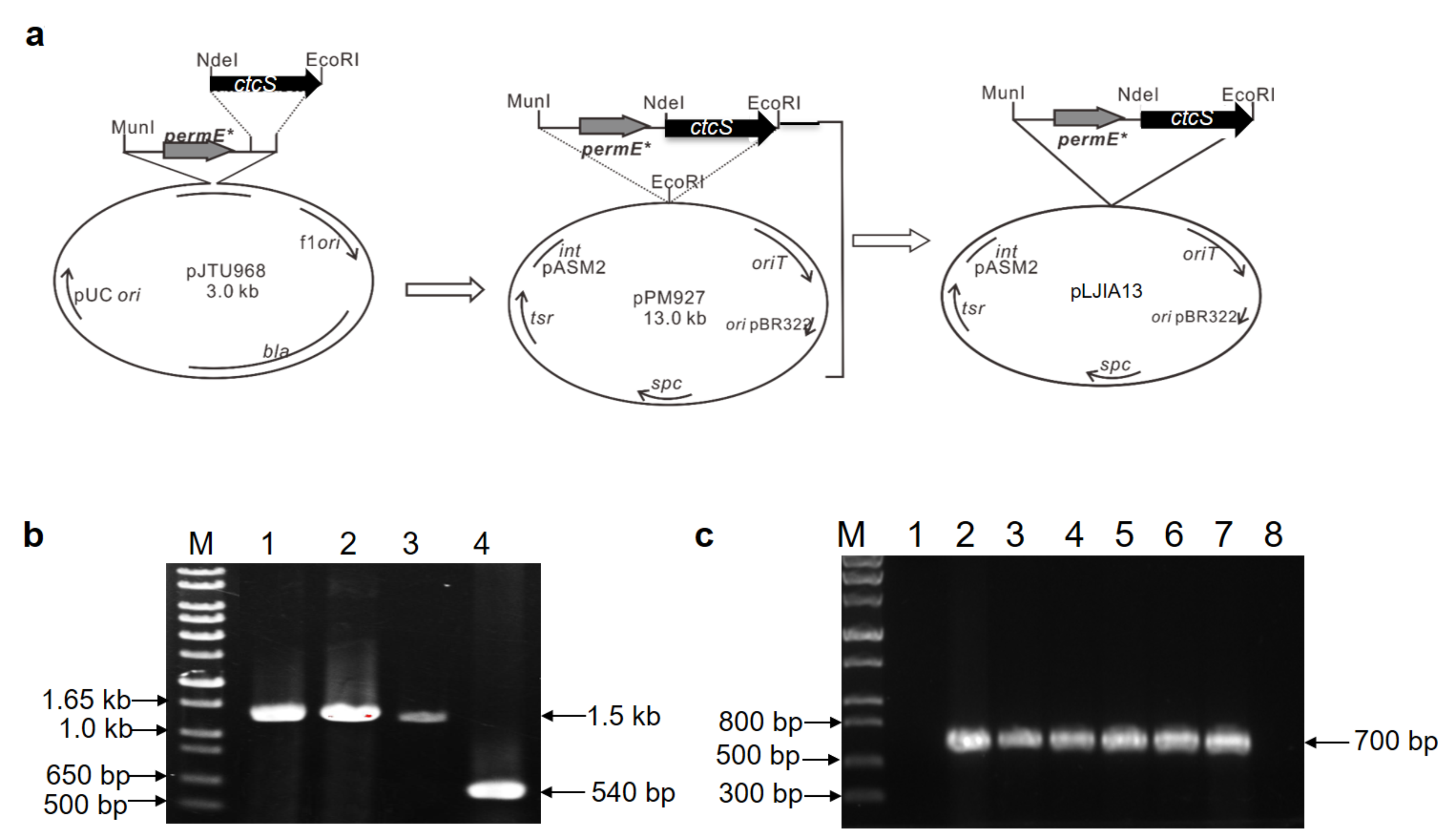

Supplement: Supplementary file 1 — Additional file 1: Figure S1. Construction and verification of the strains used in this study. (a) Schematic construction of ∆ctcS::ctcS strains. (b) PCR verification of ∆ctcS mutants. PCR products using genomic DNA from ∆ctcS mutants were in three lanes marked 1, 2 and 3. The amplified product of WT strain was used as control. (c) PCR verification of ∆ctcS::ctcS strains and WT::ctcS strains. Primers thiof-thior were used for the verification of the existence of plasmid pLJIA13 in ∆ctcS::ctcS strains (lanes marked 1, 2 and 3) and plasmid pLJIA15 in WT::ctcS strains (lanes marked 4, 5 and 6). Both of the genomic DNA of ∆ctcS strain and WT strain were used as control. [file 12866_2019_1670_MOESM1_ESM.tif]

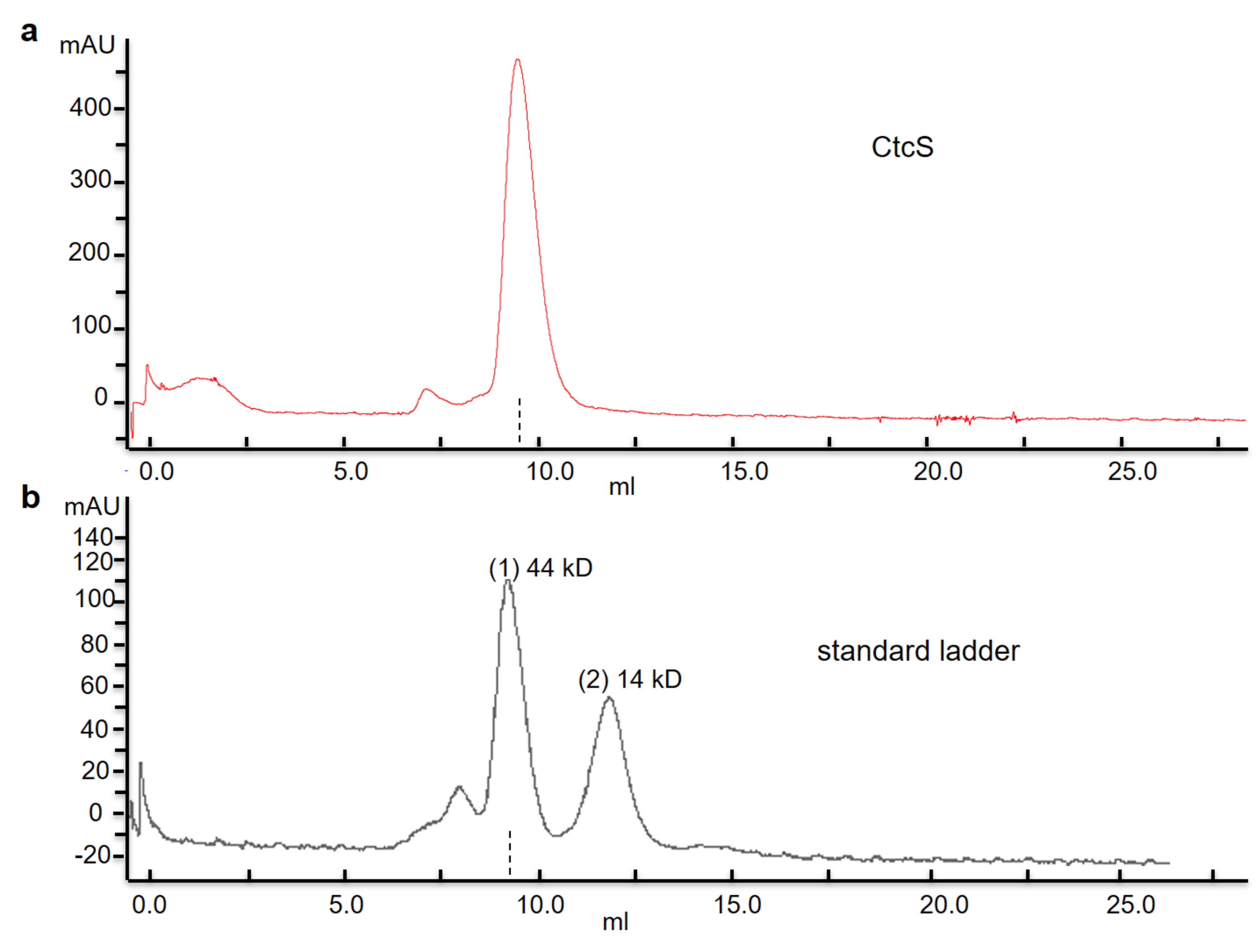

Supplement: Supplementary file 2 — Additional file 2: Figure S2. Size exclusion chromatography of His6-tagged CtcS. (a) Size exclusion chromatography analysis of purified His6-tagged CtcS. (b) Size exclusion chromatography analysis of standard ovalbumin (1) and lysozyme (2). [file 12866_2019_1670_MOESM2_ESM.tif]
