# Supplementary material for: CtcS, a MarR family regulator, regulates chlortetracycline biosynthesis
Source: BMC Microbiol. 2019 Dec 10;19:279. doi: 10.1186/s12866-019-1670-9 (PMC6905112; doi:10.1186/s12866-019-1670-9)
Supplement: Supplementary file 3 — Additional file 3: Table S1. Bacterial strains and plasmids used in this study. [file 12866_2019_1670_MOESM3_ESM.doc]

**Supplementary table S1.** **Bacterial Strains and plasmids used in this study.**

| **Strains** | **Relevant genotype** | **Source** |
| --- | --- | --- |
| *Streptomyces* | | |
| *S. aureofaciens* F3 WT strain Jinhe Biotech.Co., Ltd. (Hohhot, China) | | |
| *ΔctcS* | *ctcS* deletion mutant | This study |
| *ΔctcS::*pPM927 | *ΔctcS* strain integrated with the plasmid pPM927 | This study |
| *ΔctcS::ctcS* | *ΔctcS* complementary strain | This study |
| WT::pIB139 | *ctcS* strain integrated with the plasmid pIB139 | This study |
| WT::*ctcS* | *ctcS* overexpressing strain | This study |
| *Escherichia coli* | | |
| DH10B | General cloning host for plasmid construction | GibcoBRL |
| BL21(DE3)/plysE | Host for protein expression | Stratagene |
| BW25113 | Host for PCR-targeting manipulation | [1] |
| ET12567/pUZ8002 | Methylation-deficient intergeneric conjugation strain | [2] |
| Plasmids |  |  |
| fosmid 11D1 | Vector containing *ctc* gene cluster cluster | [3] |
| pMD18-T | TA cloning vector | TaKaRa |
| pIJ778 | *aadA*, *oriT* | [4] |
| pJTU968 | pRSETb derivative carrying strong constitutive promoter *ermE*p* | [5] |
| pPM927 | pSAM2 derivative integrative shuttle plasmid | [6] |
| pIB139 | pSET152 derivative integrative shuttle plasmid carrying *ermE*p* | [7] |
| pET-28a | Vector for protein expression in *E. coli* | Novagen |
| pLJIA03 | *ctcS* deletion plasmid based on fosmid 11D1 | This study |
| pLJIA07 | *ctcS* overexpression vector based on pET-28a in *E. coli* | This study |
| pLJIA10 | pJTU968 derivative containing intact *ctcS* digested from pLJIA07 | This study |
| pLJIA13 | pPM927 derivative containing intact *ctcS* under *ermE*p* from pLJIA10 | This study |
| pLJIA14 | pMD-18T derivative containing the intergenic region of *ctcR*-*ctcS* | This study |
| pLJIA15 | pIB139 derivative carrying *ctcS* for overexpression in WT strain | This study |

**References**

1. Datsenko K, Wanner B. One-step inactivation of chromosomal genes in *Escherichia coli* K-12 using PCR products. Proc Natl Acad Sci U S A. 2000;97(12):6640-45.

2. Paget M, Chamberlin L, Atrih A, Foster S, Buttner M. Evidence that the extracytoplasmic function sigma factor sigmaE is required for normal cell wall structure in *Streptomyces coelicolor* A3(2). J Bacteriol 1999;181(1):204–11.

3. Zhu T, Cheng X, Liu Y, Deng Z, You D. Deciphering and engineering of the final step halogenase for improved chlortetracycline biosynthesis in industrial *Streptomyces aureofaciens*. Metab Eng. 2013;19:69-78.

4. Gust B, Challis G, Fowler K, Kieser T, and Chater K. PCR-targeted *Streptomyces* gene replacement identifies a protein domain needed for biosynthesis of the sesquiterpene soil odor geosmin. Proc Natl Acad Sci U S A. 2003;100(4):1541-46.

5. Zhang, W. K., Wang, L., Kong, L. X., Wang, T., Chu, Y., Deng, Z. X. and You, D. L. Unveiling the post-PKS redox tailoring steps in biosynthesis of the Type II polyketide antitumor antibiotic xantholipin. Chem. Biol. 2012; 19(3), 422-432.

6. Smokvina T, Mazodier P, Boccard F, Thompson C, Guérineau M. Construction of a series of pSAM2-based integrative vectors for use in *Actinomycetes*. Gene. 1990;94(1):53-59.

7. Zhu T, Cheng X, Liu Y, Deng Z, You D. Deciphering and engineering of the final step halogenase for improved chlortetracycline biosynthesis in industrial Streptomyces aureofaciens. Metab Eng. 2013;19:69-78.
