# Supplementary material for: CtcS, a MarR family regulator, regulates chlortetracycline biosynthesis
Source: BMC Microbiol. 2019 Dec 10;19:279. doi: 10.1186/s12866-019-1670-9 (PMC6905112; doi:10.1186/s12866-019-1670-9)
Supplement: Supplementary file 4 — Additional file 4: Table S2. Primers used in this study. [file 12866_2019_1670_MOESM4_ESM.docx]

**Supplementary table S2.** **Primers used in this study.**

| **Primers** | **Sequence (5’-3’)** | | **Use(s)** |
| --- | --- | --- | --- |
| Gene disruption, complementation and overexpression | | | |
| targS-F | | TCCCCCGCGGCCGAGACCTTGAGTGACATCACCACGGAAATTCCGGGGATCCGTCGACC | *ctcS* gene deletion |
| targS-R | | GCCGACCGCCGCCGGGGGCAGGCCCTCGGCCAGGACCGCTGTAGGCTGGAGCTGCTTC | *ctcS* gene deletion |
| targSC-F | | AGGCTGGCCGTGGAGAGCCTG | Verification of *∆ctcS* |
| targSC-R | | GAGTACGAGGTCACGCTGGACC | Verification of *∆ctcS* |
| thiof | | 5′-ATGCGGGGATCGACCGCGCG-3′ | Confirmation of |
| thior | | 5′-TCATCAGCTGCATACCGCTG-3′ | complementation and overexpression |
|  | |  | strain |
| ctcS-F | | AAACATATGACCGATCTCTCCCCCGCGGCC | Overexpression of |
| ctcS-R | | AAAGAATTCTCACGCTGGACCTGTGACGTCGGC | *ctcS* in *E*. *coli* |
| qRT-PCR | | |  |
| ctcG-RT-F | | ACGTCGCCCTGGTGACCGGGGCGA | *ctcG*-*ctcD* operon |
| ctcG-RT-R | | GACCGTGCTCACGTCCTGCGCGGA |  |
| ctcH-RT-F | | ACCACCGACGCAACGAGGAGGCCC | *ctcH*-*ctcK* operon |
| ctcH-RT-R | | GGCGCCCTGCTCGATCGCCGCGCG |  |
| ctcM-RT-F | | CCGCCTACCTGGCCTCGCAGCCGC | *ctcM*-*ctcL* operon |
| ctcM-RT-R | | CACGATGTCGTCCACCACCAGTGA |  |
| ctcN-RT-F | | CGGGAGCGGGGCCGACCGGCCTGA | *ctcN*-*ctcP* operon |
| ctcN-RT-R | | GTTGCCGCCCTGCAGCACGCCCGC |  |
| ctcQ-RT-F | | TGCCGCTGGACCTGGCCCCCGGCC | *ctcQ* gene |
| ctcQ-RT-R | | CAGCACCCGGCTGTCGCGGTTGAT |  |
| ctcW-RT-F | | GCCGGCGCGTGGTGGTCACCGGGA | *ctcW*-*ctcT* operon |
| ctcW-RT-R | | ACGGATCTGACGCTGCGTCAATCC |  |
| ctcX-RT-F | | GCGTGGTCGGCGGCGGCCCGGCCG | *ctcX*-*ctcY* operon |
| ctcX-RT-F | | GTCGTGCTCGACGGCGCCGTGCGC |  |
| ctcR-RT-R | | ATGGCGAACGCCACCTCGCAGACCG | *ctcR* gene |
| ctcR-RT-R | | TCACTCCTCGTCGCCAGTTCCCGCA |  |
| hrdB-RT-F | | CGCTCCCGCCGGAGATCGCCGATT | Internal reference |
| hrdB-RT-R | | CGCGGCACTGACCATCAGTGTCAC |  |
| EMSA and DNase I footprinting | | | |
| ctcRS-F | | CACGGCCAACAGGAGCCCGGACAG | Cloning of intergenic |
| ctcRS-R | | CGCTGTCCGCCCGCTTCGGGCTCA | region of *ctcR*-*ctcS* |
| M13F-47 | | CGCCAGGGTTTTCCCAGTCACGAC | Probe preparation |
| M13R-48 | | AGCGGATAACAATTTCACACAGGA |  |

^a^ Restriction sites and mutation sites are underlined
